# Primary Ciliary Dyskinesia Associated Disease-Causing Variants in *CCDC39* and *CCDC40* Cause Axonemal Absence of Inner Dynein Arm Heavy Chains DNAH1, DNAH6, and DNAH7

**DOI:** 10.3390/cells13141200

**Published:** 2024-07-15

**Authors:** Alina Wilken, Inga Marlena Höben, Alexander Wolter, Niki Tomas Loges, Heike Olbrich, Isabella Aprea, Bernd Dworniczak, Johanna Raidt, Heymut Omran

**Affiliations:** 1 Department of General Pediatrics, University Hospital Muenster, 48149 Muenster, Germany; 2 Department of Psychiatry, Ruhr University Bochum, LWL University Hospital, 44791 Bochum, Germany

**Keywords:** PCD, motile cilia, ruler, IDA, diagnostics, axonemal disorganization

## Abstract

Disease-causing bi-allelic DNA variants in *CCDC39* and *CCDC40* are frequent causes of the hereditary disorder of primary ciliary dyskinesia (PCD). The encoded proteins form a molecular ruler complex, crucial for maintaining the 96 nm repeat units along the ciliary axonemes. Defects of those proteins cause a stiff, rapid, and flickery ciliary beating pattern, recurrent respiratory infections, axonemal disorganization, and abnormal assembly of GAS8, CCDC39, and DNALI1. We performed molecular characterization of the defects in the 96 nm axonemal ruler due to disease-causing variants in *CCDC39* and *CCDC40* and analyzed the effect on additional axonemal components. We identified a cohort of 51 individuals with disease-causing variants in *CCDC39* and *CCDC40* via next-generation sequencing techniques and demonstrated that the IDA heavy chains DNAH1, DNAH6, and DNAH7 are conspicuously absent within the respiratory ciliary axonemes by immunofluorescence analyses. Hence, we show for the first time that the centrin2 (CETN2) containing IDAs are also affected. These findings underscore the crucial role of CCDC39 and CCDC40 in the assembly and function of IDAs in human respiratory cilia. Thus, our data improve the diagnostics of axonemal ruler defects by further characterizing the associated molecular IDA defects.

## 1. Introduction

Primary ciliary dyskinesia (PCD) (MIM_244400) is a group of rare genetically and clinically heterogeneous disorders characterized by impaired ciliary function with a global prevalence of approximately 1:10,000 [1]. Ciliary dysfunction causes an impaired mucociliary clearance, leading to chronic upper and lower airway infections. PCD is also associated with non-respiratory disease manifestations such as laterality defects, male infertility, and, in rare cases, hydrocephalus [1,2]. So far, more than 50 PCD-associated genes have been identified [2].

**Figure 1:**
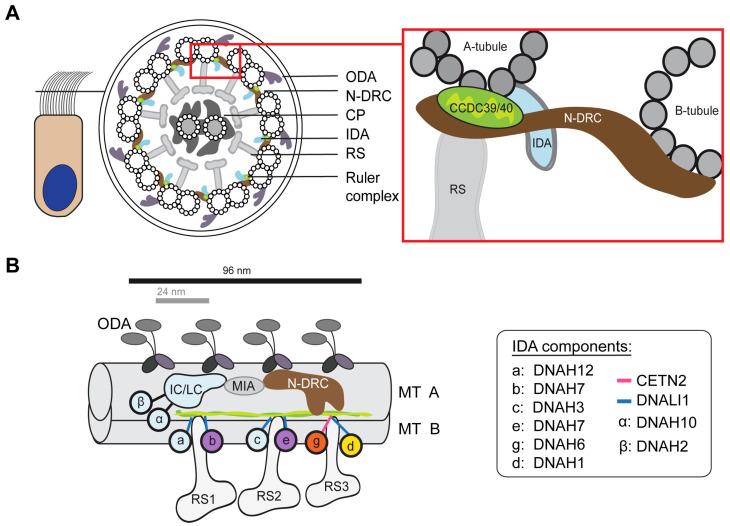
Structure of the respiratory ciliary axoneme: (**A**) Schematic cross-section of a motile respiratory cilium illustrating the 9 + 2 composition and axonemal components. The close-up on the right shows the location of the ruler complex, consisting of CCDC39 and CCDC40, the IDAs, and the N-DRC linking to the A- and B-tubule. (**B**) Schematic of the axonemal 96 nm repeat unit, including the composition of the IDA subspecies and the location of axonemal components. DNAH7, DNAH6, and DNAH1 are highlighted in purple, orange, and yellow, respectively. The stalks of the IDA dynein heavy chains are highlighted in blue or pink, depending on whether they are associated with DNALI1 or CETN2. The CCDC39/40 ruler complex is depicted in light and dark green. CP: central pair; ODA: outer dynein arm; IDA: inner dynein arm; RS: radial spoke; IC/LC: intermediate chain/light chain complex; MIA: modifier of inner arms; N-DRC: nexin–dynein regulatory complex; MT: microtubule.

Motile cilia are evolutionary highly conserved tubulin-based structures with a 9 + 2 microtubule arrangement [3,4]. Microtubule (MT) doublets, consisting of an incomplete B-microtubule at the surface of a complete A-microtubule, are interconnected by nexin–dynein regulatory complexes (N-DRC) and connected to the central pair (CP) apparatus of two inner single microtubules by T-shaped radial spoke (RS) structures (Figure 1A). The outer (ODAs) and inner dynein arms (IDAs) are motor protein complexes attached to the A tubulus and represent the key structures for proper motility [4]. One 96 nm repeat contains four ODAs, one double-headed IDA I1, which is associated with the intermediate chain/light chain (IC/LC) complex, and six single-headed IDAs [4]. Studies in *Chlamydomonas reinhardtii* revealed that each IDA complex differs in the composition of the dynein heavy chains and plays a specialized role in regulating the ciliary movement (Figure 1B) [5]. To maintain the establishment of the 96 nm repeat units along the ciliary axoneme, CCDC39 and CCDC40 form a heterodimer, which functions as a molecular ruler (Figure 1B) [1,6]. Disease-causing variants in *CCDC39* and *CCDC40* lead to the absence of the CCDC39/40 complex [7], showing the interaction of both proteins and disorganization of the 9 + 2 microtubule ultrastructure including translocated peripheral MT doublets [8,9,10,11]. Furthermore, the molecular ruler is important for the attachment of the N-DRC and the IDAs to the microtubule [6]. The loss of CCDC39 and CCDC40 causes the absence of the N-DRC component GAS8 (also known as GAS11) and the IDA light intermediate chain DNALI1 [8,9]. Due to structural abnormalities within the axoneme, affected motile cilia exhibit compromised functionality, characterized by extremely stiff, rigid, and ineffective, rapidly flickering beating patterns [8,9,11,12]. Consequently, mucociliary clearance is impaired, leading to chronic destructive airway disease. In addition, laterality defects, including situs inversus, occur in about half of affected individuals due to randomization of left/right body asymmetry [8,9]. Disease-causing variants in *CCDC39* and *CCDC40* are relatively frequent genetic causes of PCD [2,13]. Recent studies showed that affected men are also infertile due to morphological abnormalities in sperm and immotile flagella [14,15,16].

In this study, using a combined approach of panel diagnostic, whole exome sequencing, and Sanger sequencing, we identified PCD-affected individuals with disease-causing variants in either *CCDC39* or *CCDC40*. We identified five novel variants in *CCDC39* and seven novel variants in *CCDC40*. So far, the effects of ruler defects on the assembly and function of human IDA components have only been reported for the IDA light intermediate chain DNALI1 [8,9,11]. However, the effect on the CETN2 (centrin2) containing IDAg and IDA heavy chains remains unknown. In this study, we aim to define in more detail the correlation of 96 nm ruler defects and their impact on the axonemal assembly of additional IDA components. Therefore, we investigated the localization of the DNALI1-associated dynein heavy chains DNAH1 and DNAH7 and the CETN2-associated heavy chain DNAH6 in the ciliary axoneme of respiratory cells from PCD individuals with bi-allelic disease-causing variants in *CCDC39* or *CCDC40*. It is the first time the CETN2-containing IDA group has been analyzed, and in consequence, both single-headed IDA groups were systematically analyzed.

## 2. Materials and Methods

### 2.1. Human Samples and Ethics Statement

Individuals in this study were recruited from the General Pediatrics department at the University Hospital Muenster. Genomic DNA was isolated directly from blood samples using standard methods. Human samples of respiratory cilia were obtained by nasal brush biopsy and suspended in a cell culture medium (RPMI 1640, Thermo Fisher Scientific, Waltham, MA, USA). The study was approved by the Institutional Ethics Review Board at the University of Muenster. Signed informed consent was obtained from patients and/or legal guardian and their family members prior to participation.

### 2.2. Immunofluorescence (IF) Staining of Human Respiratory Epithelium

Respiratory epithelial cells were obtained by nasal brush biopsy and spread onto glass slides, air-dried, and stored at −80 °C until use. Cells were fixed with 4% paraformaldehyde and washed with Phosphate-Buffered Saline (PBS). To permeabilize the cells, 0.2% Triton-X 100 was used. Blocking was performed in 5% skim milk before incubation with primary (3–4 h at room temperature or overnight at 4 °C) and secondary (30 min at room temperature) antibodies [17]. The antibodies were diluted in 1% skim milk. Further information about the dilution of antibodies and their manufacturer is described in Table S1. DNA was stained with Hoechst33342 (Thermo Fischer Scientific, Waltham, MA, USA). The slides were mounted in a DAKO fluorescence mounting medium (Dako North America Inc., Carpinteria, CA, USA, S3023). Immunofluorescence images were taken on Zeiss Apotome Axiovert 200 (Carl Zeiss Microscopy GmbH, Jena, Germany) and processed using AxioVision 4.8 (Carl Zeiss Microscopy GmbH, Jena, Germany)and Adobe Creative Suite 4 (Adobe Systems, San José, CA, USA).

### 2.3. Western Blotting

Proteins were separated on NuPAGE 3–8% TRIS-acetate gel and transferred to the PVDF filter membrane. PVDF membranes were blocked overnight at 4 °C in 5% skim milk in TBS-T and subsequently immunoblotted with either anti-DNAH1, anti-DNAH6, and anti-DNAH7 rabbit polyclonal antibodies diluted in 5% skim milk in TBS-T (Table S1). Membranes were washed in TBS-T before incubation with the secondary goat anti-rabbit HRP antibody for 1 h at room temperature. Membranes were then washed in TBS-T and developed by using ECL Prime Western Blotting Detection Reagent (GE Healthcare Life Sciences, Chalfont St Giles, UK). Images were digitally acquired using FUSION-SL 3500WL Advance Imager (PeqLab, VWR International, Radnor, PA, USA) and modified using Adobe Creative Suite CS4.

Additional methods are provided in the Supplementary Materials.

## 3. Results

### 3.1. Identification of Bi-Allelic Disease-Causing CCDC39 Variants in PCD Individuals

**Figure 2:**
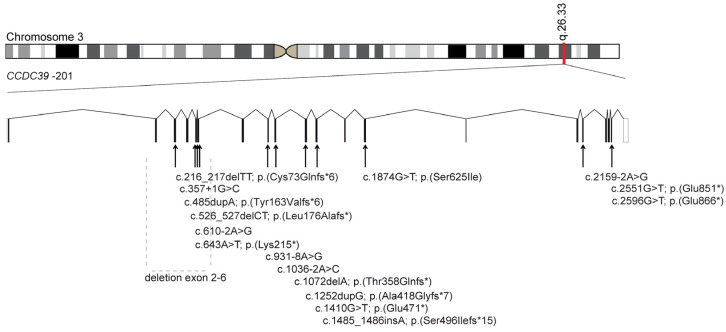
Location of the disease-causing *CCDC39* variants. *CCDC39* (NM_181426.2) is located on chromosome 3 and encodes 20 exons. In this study, we identified 17 disease-causing variants in *CCDC39*. This schematic represents the location of those variants.

*CCDC39* (NM_181426.2) is located on chromosome 3 and contains 20 exons, encoding a 941 amino acid protein. We performed whole exome sequencing and targeted PCD gene panel sequencing (Table S2) and identified disease-causing bi-allelic variants in *CCDC39* in 18 individuals from 14 unrelated families (Figure 2). The results were confirmed by Sanger sequencing. Five variants are novel to this study. The clinical manifestations in the cohort consistently included classical PCD symptoms caused by disease-causing bi-allelic *CCDC39* variants. All affected individuals showed recurrent respiratory symptoms. Laterality defects, encompassing situs inversus totalis and situs inversus abdominalis, were observed in eleven study participants (Table S3). Ciliary beating showed uncoordinated, stiff beating patterns with a reduced beating amplitude. Ten individuals carry homozygous disease-causing variants, while eight individuals carry compound heterozygous disease-causing variants. Of note, consanguinity was evident in eight families, whereas seven were non-consanguineous, or the consanguinity status remained undetermined (Table S3). Transmission electron microscopy (TEM) analyses were performed for five individuals. They revealed, in all cases, a tubular disorganization consistent with a CCDC39 deficiency [8]. All identified *CCDC39* variants were either not reported or showed only minor allele frequencies within the Genome Aggregation Database (gnomAD) [18].

### 3.2. Examination of Pathogenicity of the Novel Disease-Causing CCDC39 Variants

In this study, we identified five novel disease-causing variants in *CCDC39*. The siblings OP-2263 II1 and II2 are descended from a consanguineous family and exhibit a novel homozygous deletion of *CCDC39* exons 2 to 6. Additionally, a homozygous loss-of-function variant (c.643A>T; p.(Lys215*)) attributed to an adenine to thymine base substitution was identified in OP-567. In OP-336 II1, a novel homozygous splice site variant (c.1035-2A>G) was identified, reported with a minor allele frequency of 0.0000406 in gnomAD [18]. SpliceAI Lookup [19] predicts an acceptor splice site with a probably altered splicing effect. In the two siblings of the consanguineous family F-901, we identified a homozygous cryptic splice site variant (c.931-8A>G), which is not annotated in any public database but segregating over the family F-901. For this variant, the SpliceAI Lookup [19] predicts an intronic acceptor site with a potential altering effect on splicing. We confirmed on the cDNA level that the cryptic splice site is activated in the two siblings, leading to the insertion of seven base pairs, resulting in a frameshift and a premature stop codon (p.(Leu311*)) (Figure S1). In five unrelated families (F-651 II1, OP-964 II1, OP-1867 II1, OP-26924 II1, and OP-2690 II1), we identified the highly conserved facultative splice site variant c.1874G>T, annotated with a minor allele frequency of 0.000065 in gnomAD [18]. ClustalOmega alignment analysis [20] demonstrated that this variant is localized in an evolutionary conserved region of *CCDC39* (Figure S2A). Provean [21] databases predict the variant as deleterious. PolyPhen-2 [22] underscored its deleterious potential, with a score of 0.999 denoting probable damage. SpliceAI Lookup [19] predicts that the variant leads to an alteration of the wild-type donor site with a potential alteration of splicing. We could confirm that the splice site variant leads to retention of the intron 13 indicated by the missing PCR band in the Epstein–Barr (EB) transformed lymphocytes from whole blood of F-651 II1 (Figure S2B,C), which may result in a frameshift and premature termination of translation. In addition to this splice site variant, OP-964 II1 carries another novel frameshift variant c.1252dup, p.(Ala418Glyfs*7) on the other allele, confirmed by Segregation analysis. Furthermore, the pathogenicity of novel variants in *CCDC39* could be confirmed for the individuals F-651 II1, F-901 II0 and II1, OP-336 II, OP-736, OP-964 II1, OP-1867 II1, and OP-2624 II1 by negative CCDC39, GAS8, and/or DNALI1 immunofluorescent (IF) stainings, which confirmed that the splicing defect leads to transcript degradation (Table S4). All individuals reported in this study display nonsense, splice site, and frameshift variants predicting an early protein truncation. Therefore, according to classification guidelines of the American College of Medical Genetics and Genomics (ACMG) [23], all variants reported in this study are disease-causing.

### 3.3. Identification of Bi-Allelic Disease-Causing CCDC40 Variants in PCD Individuals

**Figure 3:**
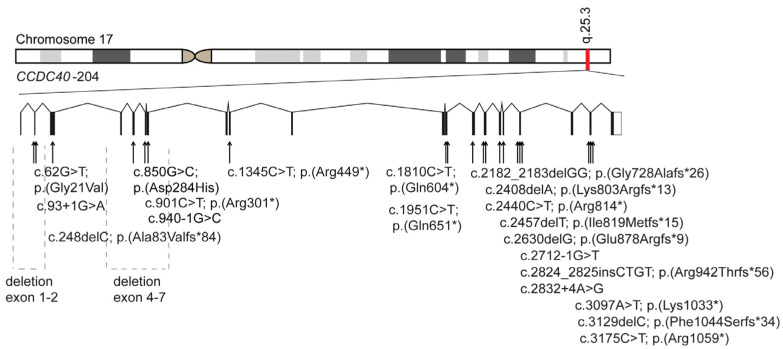
Location of disease-causing *CCDC40* variants. *CCDC40* (NM_017950.4) is located on chromosome 17 and encodes 20 exons. In this study, we identified 22 disease-causing variants in *CCDC40*. This schematic represents the location of those variants.

*CCDC40* (NM_017950.4) is located on chromosome 17, comprises 20 exons, and encodes a protein of 1142 amino acids (Figure 3). Our study includes 33 PCD individuals of 29 unrelated families with disease-causing variants in *CCDC40,* identified through a combined approach of whole exome sequencing and targeted PCD gene panel diagnostics. All variants underwent verification via Sanger sequencing, and if possible, mutational status was investigated using segregation analyses. Eight families reported consanguinity, whereas 14 were non-consanguineous, and for eleven families, the consanguinity status is unknown (Table S5). All individuals with bi-allelic disease-causing variants in *CCDC40* included in this study suffer from typical PCD symptoms, confirming the pathogenicity for all reported *CCDC40* variants according to the ACMG guidelines [23]. Situs inversus was reported in eleven individuals, while 14 showed no laterality defects, and situs status remained unknown for eight individuals. High-speed video microscopy analyses (HVMA) showed the typical beating pattern of CCDC40 deficient cilia with stiff, rigid, and rapid flickery movements. TEM analyses in five individuals revealed ciliary cross-sections exhibiting tubular disorganization (Table S5).

### 3.4. Examination of Pathogenicity for the Novel Disease-Causing CCDC40 Variants

We identified 22 distinct variants, including seven novel disease-causing variants in *CCDC40* (NM_017950.4). All variants are extremely rare, with minor or absent allele frequency, according to gnomAD [18]. The most prevalent variant, *CCDC40* c.248del; p.(Ala83Valfs*84) (rs397515393), was found in 18 of 33 individuals in a homozygous or compound heterozygous state. Despite its recurrence in our cohort, this variant showed a minor allele frequency of 0.000563, according to gnomAD [18]. IF staining revealed abnormal localization of CCDC39, GAS8, and/or DNALI1 in all individuals, confirming the pathogenicity of all identified variants (Table S6). Individual OP-82 harbors a compound heterozygous variant comprising the frameshift variant and a novel nonsense variant, *CCDC40* c.1345C>T; p.(Arg449*). Additionally, we identified a novel homozygous missense variant in *CCDC40* c.62C>T, p.(Gly21Val) in the siblings OP-2072 II2 and II4. Consanguinity likely explains this homozygosity, although parental DNA was not available. This variant is not annotated in gnomAD [18]. Provean [21] predicts the amino-acid exchange as neutral. ClustalOmega [20] alignment analysis shows that this variant is not localized in an evolutionary conserved region of *CCDC40* (Figure S3A). Due to the additional diagnostic results, tubular disorganization, and translocation of the peripheral MT doublets in TEM; stiff ciliary beating pattern in HVMA; and abnormal localization of the proteins GAS8, CCDC39, and DNALI1 in immunofluorescence staining, we believe that this variant is altering the protein function (Figure S3). Furthermore, we identified the novel frameshift variant *CCDC40* c.2408del; p.(Lys803Argfs*13) in the siblings OP-2242 II1 and OP-2302 II1 from a non-consanguineous family. They carry this variant compound heterozygous with the nonsense variant *CCDC40* c.901C>T, p.(Arg301*) (rs201223986). Due to the lack of parental DNA, segregation analysis was not performed. We could demonstrate by IF analyses that CCDC39 and GAS8 showed an abnormal localization in respiratory ciliary axonemes and tubular disorganization by TEM, supporting the pathogenicity. Additionally, we detected two large deletions in unrelated individuals: OI-101 exhibited a homozygous deletion of *CCDC40* exon 4 to 7, while OP-1072 II1 carried a homozygous deletion of *CCDC40* exon 1 to 2. Both individuals displayed abnormal CCDC39, GAS8, and DNALI1 localization by IF analyses (Table S6) and tubular disorganization by TEM. Furthermore, we identified a novel homozygous frameshift variant *CCDC40* c.2630del, p.(Glu878Argfs*9) in OP-1261 II1. For OP-1854 II1, we could also identify a novel splice site variant *CCDC40* c.93+1G>A. Both individuals show an absence of CCDC39 and GAS8 by IF stainings. For the donor splice site variant c.93+1G>A, SpliceAI Lookup [19] predicts a pathogenic effect of splicing with a high probability. Consequently, based on ACMG criteria and our findings, all variants identified in *CCDC40* are disease-causing [23].

### 3.5. Defects of the 96 nm Axonemal Ruler Cause Deficiency of IDA Heavy Chains DNAH1, DNAH6, and DNAH7

**Figure 4:**
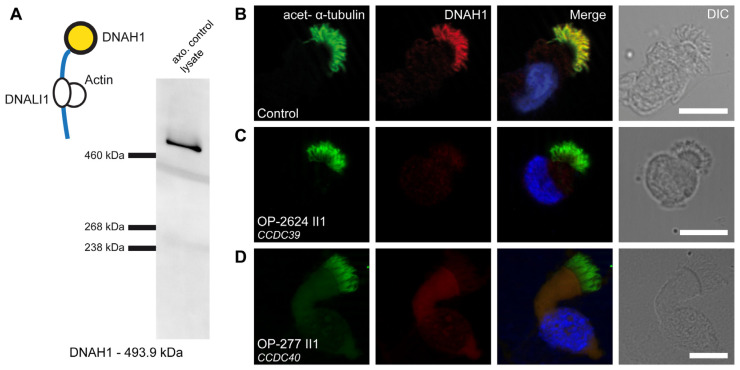
DNAH1 is absent from axonemes of respiratory cilia from PCD individuals carrying disease-causing *CCDC39* and *CCDC40* variants: (**A**) DNAH1 forms the heavy chain of IDA d and is associated with DNALI1. We detected a specific band for DNAH1 in the axonemal control lysate of respiratory cells at the expected size of ~494 kDa by WB. (**B**) Respiratory cilia double-labeled with antibodies directed against acetylated α-tubulin (green) and DNAH1 (red) show co-localization of DNAH1 with acetylated α-tubulin along the cilia from unaffected controls (yellow). (**C**,**D**) In contrast, DNAH1 is absent or severely reduced in the PCD individuals OP-2624 II1 and OP-277 II1 carrying disease-causing variants in *CCDC39* and *CCDC40*, respectively. Nuclei were stained with Hoechst33342 (blue). The scale bar represents 10 µm.

To describe the subcellular localization of the IDA heavy chains DNAH1, DNAH6, and DNAH7 within the respiratory ciliary axoneme of PCD individuals carrying disease-causing variants in *CCDC39* and *CCDC40*, we performed IF staining of 24 individuals, of whom 17 carry disease-causing variants in *CCDC40* and seven in *CCDC39*. Using Western blot (WB) analysis, we confirmed that DNAH1 is an axonemal component of respiratory cilia and furthermore the specificity of the used antibody. We could detect a single specific band at the expected size of DNAH1 around 494 kDa (Figure 4A). IF staining against DNAH1 demonstrated the localization along the full-length ciliary axoneme. The yellow color in the merged image (Figure 4B) confirmed the co-localization of DNAH1 with acetylated α-tubulin, used as a ciliary marker. In contrast, DNAH1 was absent in the respiratory ciliary axonemes from the CCDC39 deficient individual OP-2624 II4 (Figure 4C) and CCDC40 deficient individual OP-277 II1 (Figure 4D). Furthermore, also other analyzed PCD individuals with variants in *CCDC39* (*n* = 5) and *CCDC40* (*n* = 16) showed the absence of DNAH1 from respiratory ciliary axonemes (Tables S4 and S6).

**Figure 5:**
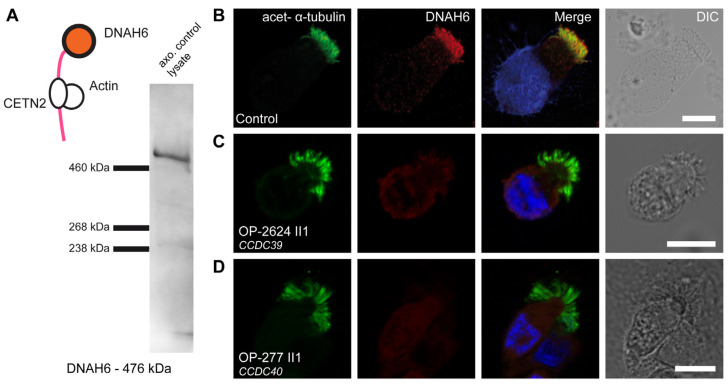
DNAH6 is absent from axonemes of respiratory cilia from PCD individuals carrying disease-causing *CCDC39* and *CCDC40* variants: (**A**) DNAH6 forms the heavy chain of IDA g and is associated with CETN2 (centrin2). We detected a specific band for DNAH6 in the axonemal control lysate of respiratory cells at the expected size of ~476 kDa by WB. (**B**) Respiratory cilia double-labeled with antibodies directed against acetylated α-tubulin (green) and DNAH6 (red) show co-localization of DNAH6 with acetylated α-tubulin along the cilia from unaffected controls (yellow). (**C**,**D**) In contrast, DNAH6 is absent or severely reduced in the PCD individuals OP-2624 II1 and OP-277 II1 carrying disease-causing variants in *CCDC39* and *CCDC40*, respectively. Nuclei were stained with Hoechst33342 (blue). The scale bar represents 10 µm.

DNAH6, the dynein heavy chain of IDAg, is connected with the light intermediate chain of IDAd. DNAH6 is associated with CETN2. We could detect a specific band of the expected size of 476 kDa by WB analyses, demonstrating the specificity of the used anti-DNAH6 antibody and confirming that DNAH6 is an axonemal component of respiratory cilia (Figure 5A). Human respiratory cilia from 4 PCD individuals with disease-causing variants in *CCDC39*, from 13 PCD individuals with disease-causing variants in *CCDC40,* and from healthy donors were stained for DNAH6 (Tables S4 and S6). Notably, while DNAH6 was localized throughout the full-length ciliary axoneme of control cells (Figure 5B), in PCD individuals with *CCDC39* and *CCDC40* variants, DNAH6 localization within the ciliary axoneme was substantially reduced or absent (Figure 5C,D).

**Figure 6:**
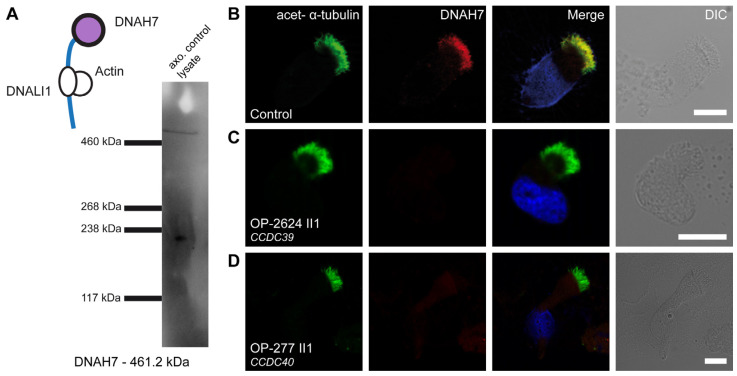
DNAH7 is absent from axonemes of respiratory cilia from PCD individuals carrying disease-causing *CCDC39* and *CCDC40* variants: (**A**) DNAH7 forms the heavy chain of IDA b and e and is associated with DNALI1. In WB, we could detect a specific band for DNAH7 in the axonemal control lysate of respiratory cells at the expected size of ~461 kDa. (**B**) Respiratory cilia double-labeled with antibodies directed against acetylated α-tubulin (green) and DNAH7 (red) show co-localization of DNAH7 with acetylated α-tubulin along the cilia from unaffected controls (yellow). (**C**,**D**) In contrast, DNAH7 is absent or severely reduced in the PCD individuals OP-2624 II1 and OP-277 II1 with disease-causing variants in *CCDC39* and *CCDC40,* respectively. Nuclei were stained with Hoechst33342 (blue). The scale bar represents 10 µm.

*DNAH7* is the only gene that encodes for two of the six heavy chains of single-headed IDAs. The protein is a component of IDAb and IDAe and is associated with DNALI1. By WB analysis of control samples, we detected a band of the expected protein size of DNAH7 (approximately 461 kDa) and hereby confirmed that DNAH7 is a component of the ciliary axoneme (Figure 6A). The localization of DNAH7 in respiratory axonemes was analyzed in seven PCD individuals with disease-causing variants in *CCDC39* and in 14 PCD individuals with disease-causing variants in *CCDC40* (Tables S4 and S6). In healthy control subjects, DNAH7 is localized throughout the entire length of ciliary axonemes (Figure 6B). PCD individuals carrying disease-causing variants in either *CCDC39* or *CCDC40* (Figure 6C,D) showed the absence of DNAH7 from the ciliary axoneme.

Additionally, we examined the localization of the three IDA heavy chains DNAH1, DNAH6, and DNAH7 in individuals with isolated N-DRC defects (OP-59 II1, OP-835 II5, and OP-1627) and ODA defects (OP-80 II4). In contrast to defects of the 96 nm axonemal ruler caused by disease-causing variants in *CCDC39* or *CCDC40*, PCD individuals with variants in *DRC1/CCDC164*, *CCDC65*, *GAS8*, encoding components of the N-DRC, or *DNAH5* encoding an ODA heavy chain, showed a normal distribution along the ciliary axoneme for all three IDA heavy chains DNAH1, DNAH6, and DNAH7 (Figure S4).

## 4. Discussion

The comprehension of the molecular mechanisms underlying ciliary function is fundamental for the treatment of PCD. The pathogenic mechanism, the clinical manifestations, and the extent of the disease depend on the affected gene [1]. Advancing our understanding of ciliary function and structures is essential to elucidate the molecular interactions and to interpret the roles of distinct axonemal components in order to enable targeted therapy for the individual PCD patient. Therefore, our objective is to further delineate defects in the 96 nm axonemal ruler, elucidate their correlations, and analyze their effect on additional axonemal components. CCDC39 and CCDC40 are evolutionarily conserved coiled-coil domain-containing proteins and are essential to ensure the correct establishment of the 96 nm repeats along the ciliary axoneme by forming a ruler complex [6]. This complex provides anchoring sites for IDA and N-DRC proteins, such as DNALI1 and GAS8/GAS11 [6,13]. The loss of the 96 nm ruler consequently leads to the complete absence of any recognizable 96 nm repeats, including the CCDC39/40 coiled coil, the loss of DNALI1 and GAS8, and is additionally associated with the structural disorganization of the axoneme and molecular transposition [7,8,9]. These findings are in line with our abnormal IF results for CCDC39, DNALI1, and GAS8 and TEM analyses. RSs are present within the axoneme but bind to the MT only sporadically, while ODAs are not affected [7,10], which could be confirmed by our normal DNAH5 IF staining. Studies in *Chlamydomonas* revealed that single-headed IDAs differ in their dynein heavy chain and contain either CETN2 (IDAg) or DNALI1 (IDAa, b, c, e, d) [5,7,24]. Each IDA type is found once within the 96 nm interval and is not functionally interchangeable [5,7]. In contrast to *Chlamydomonas*, the human orthologue DNAH7 is expected to appear in two single-headed IDA types [7]. Nevertheless, defects of the IDAs were usually characterized by the absence of DNALI1, and the localization of the IDA dynein heavy chains has never been investigated within CCDC39/40 deficient cilia. In this study, we demonstrated that CCDC39/40 deficiency not only results in abnormalities of DNALI1 but also affects the encoded heavy chains DNAH1 and DNAH7 of IDAd and IDAb/e, respectively. In addition, we could show for the first time that deficiency of CCDC39/40 also causes a loss of the CETN2 containing IDAg, including its dynein heavy chain DNAH6. Conversely, we revealed that individuals with defects of the ODAs or N-DRCs do not exhibit altered locations of the IDA heavy chains. This supports the idea that CCDC39 and CCDC40 are not only crucial to determine the ciliary 96 nm repeat units [11] but also demonstrate the putative role of CCDC39/40 ruler in tethering DNALI1- and CETN2-containing IDAs, including their heavy chains, along the whole axoneme. In addition, Brody et al. reported that proteomic analysis of ciliary protein extracts from *CCDC39*-mutant cells revealed the axonemal absence of IDA proteins, including DNAH1, DNAH6, and DNAH7 [25], which is consistent with our IF data. IDAs are responsible for regulating the waveform of ciliary beating by converting ATP into mechanical force to drive ciliary motility [5,24]. Nevertheless, there is only limited evidence suggesting that isolated IDA defects can cause PCD [4]. However, defects of the IDAs are frequently linked to male infertility and morphological abnormalities of the sperm flagella (MMAF) [4]. Disease-causing variants in the genes *DNAH1* [26], *DNAH6* [27], and *DNAH7* [28] have been described to result in severe impairment of sperm motility. In contrast, *CCDC39* and *CCDC40* are linked to PCD and MMAF, including severe tubular disorganization of the axoneme in both cell types [14,15,16]. Disease-causing *CCDC39* and *CCDC40* variants result in considerably impaired lung function, which is more pronounced than in other genotypes associated with PCD [2,13,29]. This is currently not explained. However, we can hypothesize. The ruler proteins CCDC39 and CCDC40 are essential for the correct assembly of multiple axonemal components and the structural organization of the axoneme. Disease-causing variants in *CCDC39* and *CCDC40* lead to combined defects of the 96 nm repeat units comprising IDAs, N-DRCs, and RSs, which are critical components for proper ciliary function [6,7,10]. Thus, the genetic defects affect multiple distinct protein complexes. In contrast, most other PCD variants only affect the composition of a single or few multiprotein complexes (e.g., disease-causing variants in *DNAH5*, *DNAH9,* or *DNAH11* [30,31,32]). Defects resulting in alterations of many multiprotein complexes might possibly result in a more severe clinical phenotype. In most PCD variants, ciliary beating is characterized by immotility and flaccid cilia (e.g., DNAH5 defects [30]). In those defects, mucus is probably not trapped in cilia, and thus, cough clearance is not affected. In contrast, HVMA of CCDC39 and CCDC40 deficient respiratory cilia have revealed that these structural defects result in extremely stiff and rapid ciliary beating patterns [2,8,9,12]. Possibly, the mucus may become trapped in the stiff cilia of individuals with disease-causing *CCDC39* and *CCDC40* variants, which results in reduced cough clearance. However, this has not been studied so far. Since *CCDC39* and *CCDC40* are expressed in the nodal cilia of mouse and human embryos [8,9], situs abnormalities are expected to occur in 50% of the cases and were reported in our cohort in 52%. Nearly all of the identified variants are nonsense, splice site, or frameshift variants, leading to an early protein truncation except for the amino acid exchange in *CCDC40* p.(Gly21Val). For both genes, the identified variants range over all exons. Hence, no specific cluster between the locations of disease-causing variants along the transcripts and their effects on protein structure and function was observed. This is in accordance with other studies describing that all previously published variants are distributed across the two genes, suggesting that a protein termination at any point leads to a deleterious function [11].

In conclusion, it is described that CCDC39 and CCDC40 deficient individuals have worse lung function that declines more rapidly [2,13] compared to other PCD genotypes. This can be attributed to the combined axonemal defects of several components. IDAs and N-DRCs are axonemal components that are essential for proper cilia function. Both are functionally disrupted by defects of CCDC39 and CCDC40, in addition to the loss of the 96 nm repeat units [7]. Approximately half of the affected individuals exhibit laterality defects [8,9], while males are additionally affected by infertility due to immotile sperm flagella with morphological abnormalities [14,15,16]. For the first time, we demonstrate that the defects of the molecular ruler not only cause defects of DNALI1 containing IDAs and the associated heavy chains DNAH1 and DNAH7 but also affect the CETN2 containing IDAg, including its heavy chain DNAH6. Notably, the use of antibodies directed against DNAH1, DNAH6, and DNAH7 in IF microscopy provides additional tools for the characterization of other molecular defects in CCDC39/40 deficient cilia. These results represent a significant advantage in PCD diagnostics, particularly because no specific antibody against CCDC40 is available. Especially in the case of variants of uncertain significance, this offers improved possibilities for determining the pathogenicity of the variant. Our findings not only represent a significant advancement in the diagnosis of PCD related to ruler defects but will also improve patient counseling. Affected individuals will benefit from these results for early and appropriate treatment.

## Data Availability

All data supporting this study are available in the main text or in the Supplementary Materials and on request from the corresponding authors. Primer sequences for Sanger sequencing were not included in the manuscript but are fully available from the corresponding author upon request.

## Supplementary Materials

The following supporting information can be downloaded at: https://www.mdpi.com/article/10.3390/cells13141200/s1, Figure S1: Analysis of the *CCDC39* variant in family F-901; Figure S2: Analysis of the variant *CCDC39* c.1874G>T; Figure S3: Analysis of the family OP-2072 with the homozygous variant *CCDC40* c.62C>T; p.(Gly21Val); Figure S4: Individuals with isolated nexin-link and ODA defects show a normal distribution of IDA proteins DNAH1, DNAH6, and DNAH7 along respiratory cilia; Table S1: Antibodies and dilutions, which were used for immunostaining and western blot analyses; Table S2: Table presents genes, which are included in targeted PCD gene panel sequencing; Table S3: Clinical characteristics of individuals with pathogenic bi-allelic variants in *CCDC39* (NM_181426); Table S4: Overview of the IF stainings in individuals with *CCDC39* variants and allele frequency of the identified *CCDC39* variants; Table S5: Clinical characteristics of individuals with pathogenic bi-allelic variants in *CCDC40* (NM_017950); Table S6: Overview of the IF stainings in individuals with *CCDC40* variants and allele frequency of the identified *CCDC40* variants.
