# Supplementary material for: Primary Ciliary Dyskinesia Associated Disease-Causing Variants in CCDC39 and CCDC40 Cause Axonemal Absence of Inner Dynein Arm Heavy Chains DNAH1, DNAH6, and DNAH7
Source: Cells. 2024 Jul 15;13(14):1200. doi: 10.3390/cells13141200 (PMC11274998; doi:10.3390/cells13141200)
Supplement: Supplementary file 1 [file cells-13-01200-s001.zip › cells-3014467-supplementary.pdf]

Manuscript

Title:

Primary Ciliary Dyskinesia associated disease-causing variants in *CCDC39* and *CCDC40* cause axonemal absence of inner dynein arm heavy chains DNAH1, DNAH6, and DNAH7

Authors:

Alina Wilken[1], Inga Marlena Höben[1], Alexander Wolter[2], Niki Tomas Loges[1], Heike Olbrich[1], Isabella Aprea[1], Bernd Dworniczak[1], Johanna Raidt[1], Heymut Omran[1]

[1]Department of General Pediatrics, University Hospital Muenster, Muenster, Germany; [2]Department of Psychiatry, Ruhr University Bochum, LWL University Hospital, Bochum, Germany

Supplementary Materials

Supplemental methods

High-speed video microscopy analysis (HVMA) for ciliary beat assessment in human epithelial respiratory cells

Respiratory epithelial cells were visualized with a Zeiss AxioVert A1 microscope (40x and 63x phase contrast objective) equipped with a Basler scA640-120fm monochrome high-speed video camera (Basler, Ahrensburg, Germany) set at 120 frames per second. Ciliary beat frequency and beating pattern (evaluated on slow-motion playbacks) were assessed with the Sisson-Ammons Video Analysis (SAVA) system (Ammons Engineering, Clio, USA) [12].

Transmission Electron Microscopy of Human Respiratory Cilia

Transmission electron microscopy (TEM) of human respiratory cilia was performed on human respiratory cells obtained by nasal brush biopsy or after cultivation via air-liquid interface (ALI) cell culture. Cells were fixed and prepared as previously described [16]. Images were taken with the Philips CM10 transmission electron microscope combined with a Quemesa camera and the iTEM SIS image acquisition software (both from Olympus Soft Imaging Solutions). Image processing was carried out utilizing Adobe Creative Suite CS5.

Preparation of Human Respiratory Cell Lysates

Protein extracts were prepared from human respiratory cells (either from nasal brush biopsies or spheroid cultures). Cells were incubated in 800-1000 µl NP40 or RIPA lysis buffer containing protease inhibitor cocktail (P8340, Sigma-Aldrich, St. Louis, Missouri, USA) on ice for 30 min with occasional vortexing. Lysates were centrifuged at 14,000 rpm and 4°C for 10 min. Supernatants were removed into new collection tubes (cytoplasmic fraction). Pellets were resuspended and incubated in 100-150 µl modified Reeds high salt extraction lysis buffer (30 mM HEPES pH 7.4, 5 mM $MgSO_4$, 0.1 mM EDTA, 625 nM NaCl, 2 mM DTT, 70 mM β-Mercaptoethanol, 0.1% Triton-X 100) containing a protease inhibitor cocktail (P8340, Sigma-Aldrich, St. Louis, Missouri, USA) on ice for 30-60 min and frequently vortexed. Lysates were centrifuged at 14,000 rpm at 4°C for 10 min. Supernatants were removed into new collection tubes (axonemal fraction). The protein content of the lysates were evaluated by silver staining using the ProteoSilver silver staining kit (Sigma-Aldrich, St. Louis, Missouri, USA). Lysates were stored at -20°C or -80°C until use. The axonemal fraction was used for further analysis.

Analyses of genetic variants in PCD individuals

Targeted-exome sequencing of genomic DNA was performed at the Cologne Center for Genomics (CCG). For enrichment, the NimbleGen SeqCap EZ Human Exome Library v2.0 was used and enriched preparations were sequenced with the HiSeq2000 platform (Illumina, San Diego, USA). The genome sequence hg38 was used as reference to map sequencing reads that passed quality filtering. For the diagnostic PCD gene panel DNA samples were enriched with SureSelect™ Target Enrichment Kit (Agilent Technologies, Santa Clara, USA) using the NextSeq500 Illumina® platform (Illumina, San Diego, USA). A PCD associated gene panel (Table S2) was used to execute a library preparation. Library construction was performed as described by the manufacturer and output sequencing files were evaluated with bioinformatics tools from the galaxy platform 12 (https://usegalaxy.org/). Allele frequencies of the DNA variants were obtained from the Genome Aggregation Database [18].

According to the American College of Medical Genetics and Genomics (ACMG) and the Association for Molecular Pathology (AMP) guidelines [23] only DNA variants classified as pathogenic (class 5) or likely pathogenic (class 4) were referred to as disease-causing variants. All identified variants were confirmed by Sanger-Sequencing and segregation analysis was performed from parents and/or siblings if genomic DNA was available. PCR-products were verified by agarose gel electrophoresis, purified by PCR product pre-sequencing kit (USB, Ohio, USA) and sequenced bi-directionally using BigDye Terminator v3.1 Cycle Sequencing Kit (Applied Biosystems, California, USA). Samples were separated and analyzed on an Applied Biosystems 3730xl DNA Analyzer. The resulted trace files of the Sanger sequencing are analyzed with CodonCode aligner (v.3.7.1). According to the ACMG guidelines, the novel missense variant *CCDC40* c.62C>T; p.(Gly21Val) was classified as pathogenic based on further diagnostic results including high-speed video-, transmission electron-, and immunofluorescence microscopy analyses (see Figure S3) [23].

Because no fresh biopsy material of the airways was available, we performed analyses of two splice site variants (*CCDC39* c.931-8A>G and *CCDC39* c.1874G>T) using cDNA reverse transcribed from RNA isolated from lymphocyte cultures after Epstein-Barr transformation. RNA was isolated using the RNeasy Mini Kit (Qiagen, Venlo, Netherlands). First-strand cDNA was synthesized with oligo-d(T)20 primer (Invitrogen, Carlsbad, USA) and SuperScriptIII reverse transcriptase (Invitrogen, Carlsbad, USA). First-strand cDNA was synthesized with oligo-d(T)20 primer (Invitrogen, Carlsbad, USA) and SuperScriptIII reverse transcriptase (Invitrogen, Carlsbad, USA). Nested-PCR was performed with two sets of primers, the second nested primer pair amplifying the intended product in between the first amplification minimizing non-specific products. The PCR-products were then processed for Sanger sequencing.

Supplemental figures

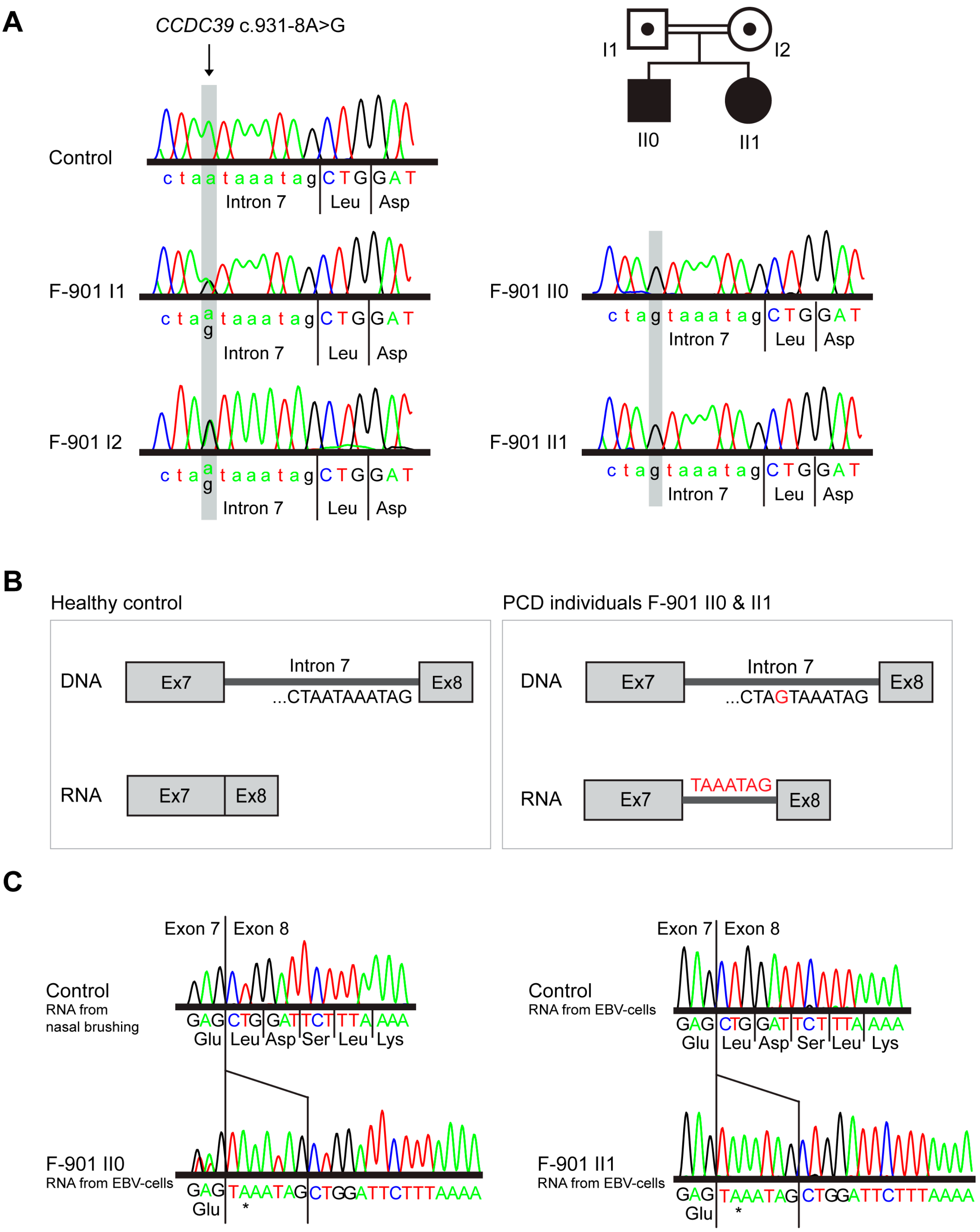

Figure S1. Analysis of the *CCDC39* variant in family F-901: (**A**) Pedigree of the consanguineous PCD family F-901. The cryptic splice site variant *CCDC39* c.931-8A>G is localized in the intronic sequence before exon 8. The segregation analysis is consistent with an autosomal recessive inheritance. (**B**) Schematic, showing the splicing defect in F-901 II0 and II1 compared to control transcript due to homozygous *CCDC39* c.931-8A>G variant. (**C**) Splice site verification reveals an insertion of 7 bp between exon 7 and exon 8 in the two affected individuals leading to a premature protein termination (p.Leu311*).

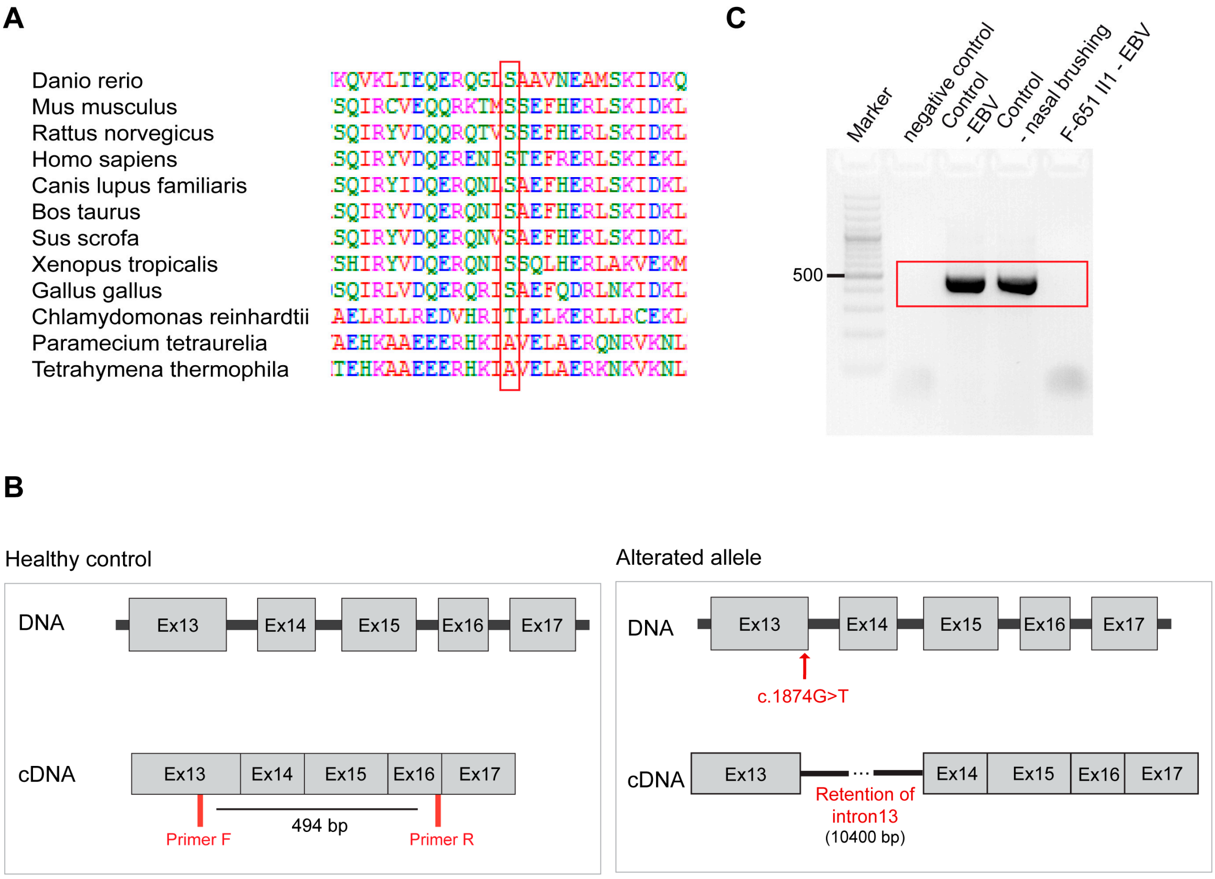

Figure S2. Analysis of the variant *CCDC39* c.1874G>T: (**A**) The variant *CCDC39* c.1874G>T leads to an amino acid exchange at position 625 (position marked by the red box). ClustalOmega alignment analysis shows that this variant is localized in an evolutionary conserved region of *CCDC39.* (**B**) Schematic showing the effect of the facultative splice site variant c.1874G>T leading to the retention of the intron between exon 13 and 14. (**C**) Agarose gel shows no amplification of the 494 bp product due to large size in comparison to the two healthy controls.

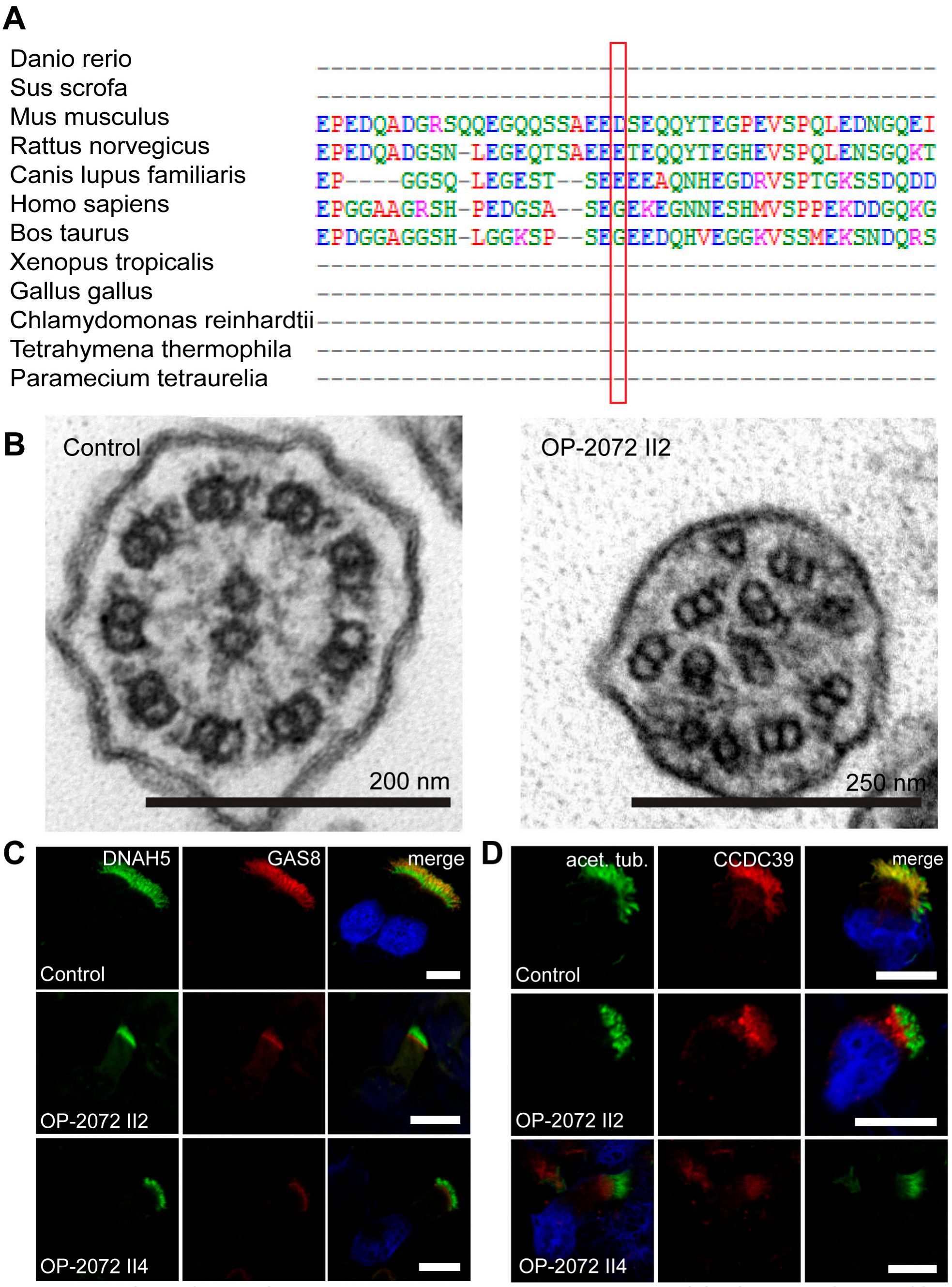

Figure S3. Analysis of the family OP-2072 with the homozygous variant *CCDC40* c.62C>T; p.(Gly21Val): (**A**) ClustalOmega alignment analysis shows that the variant *CCDC40* c.62C>T; p.(Gly21Val) is not localized in an evolutionary conserved region of *CCDC40.* (**B**) Transmission electron microscopy photographs of cross-sections through respiratory epithelial cilia demonstrate tubule disorganization. Scale bars represents 200 nm or 250 nm as indicated. (**C**) Respiratory cilia double-labeled with antibodies directed against DNAH5 (green) and GAS8 (red) show co-localization of GAS8 with DNAH5 along the cilia from unaffected controls (yellow). In contrast, GAS8 is absent or severely reduced in the PCD individuals OP-2072 II2 and OP-2072 II4. Nuclei were stained with Hoechst33342. Scale bars represent 10 µm. (**D**) Respiratory cilia double-labeled with antibodies directed against acetylated α-tubulin (green) and CCDC39 (red) show co-localization of CCDC39 with acetylated α-tubulin along the cilia from unaffected controls (yellow). In contrast, CCDC39 is absent or severely reduced in PCD individuals OP-2072 II2 and OP-2072 II4. Nuclei were stained with Hoechst33342. Scale bar represents 10 µm.

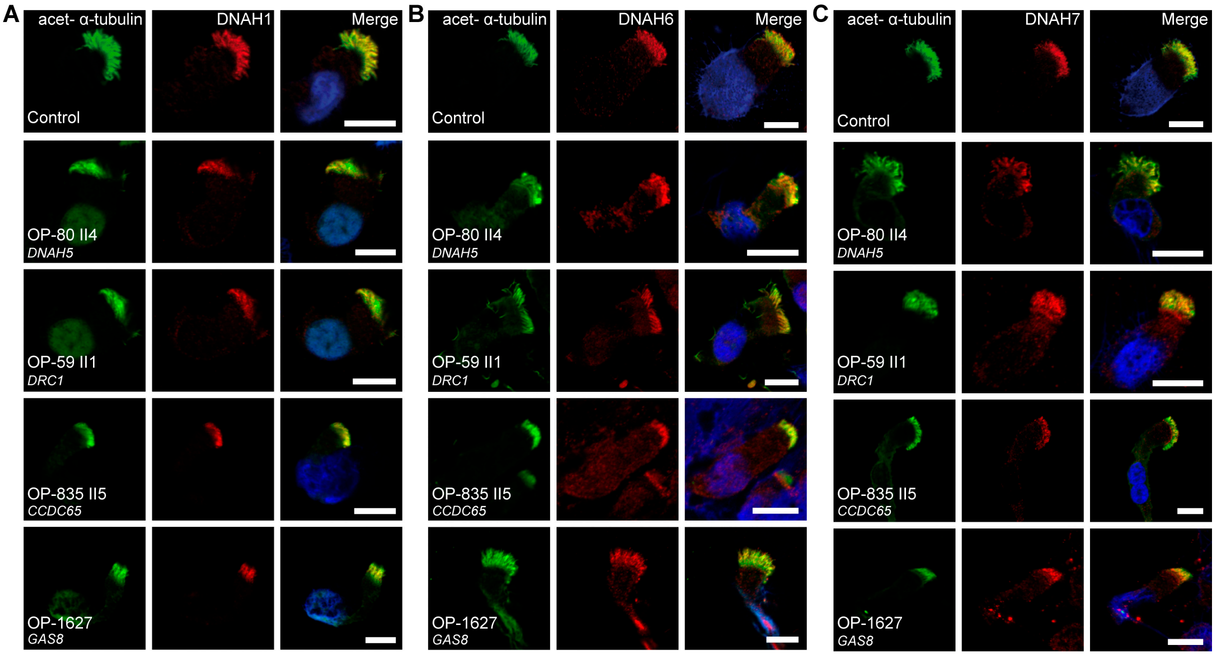

Figure S4. Individuals with isolated nexin-link and ODA defects show a normal distribution of IDA proteins DNAH1, DNAH6, and DNAH7 along respiratory cilia: (**A**) Respiratory cilia double-labeled with antibodies directed against acetylated α-tubulin (green) and DNAH1 (red), show co-localization of DNAH1 with acetylated α-tubulin along the cilia from unaffected controls (yellow) and from PCD individuals with variants in *DNAH5*, *DRC1/CCDC164*, *CCDC65*, and *GAS8/GAS11*. (**B**) Respiratory cilia double-labeled with antibodies directed against acetylated α-tubulin (green) and DNAH6 (red), show co-localization of DNAH6 with acetylated α-tubulin along the cilia from unaffected controls (yellow) and from PCD individuals with variants in *DNAH5*, *DRC1/CCDC164*, *CCDC65*, and *GAS8/GAS11*. (**C**) Respiratory cilia double-labeled with antibodies directed against acetylated α-tubulin (green) and DNAH7 (red), show co-localization of DNAH7 with acetylated α-tubulin along the cilia from unaffected controls (yellow) and from PCD individuals with variants in *DNAH5*, *DRC1/CCDC164*, *CCDC65* and *GAS8/GAS11*. All scale bars represent 10 µm. Nuclei were stained with Hoechst33342.

Supplemental Tables

Table S1. Antibodies and dilutions, which were used for immunostaining and western blot analyses.

Antibodies	Manufacturer	Order number	Dilution	
			IF	WB
Mouse monoclonal anti-acetylated α tubulin	Sigma-Aldrich	T6793	1:10,000	-
Rabbit polyclonal anti-CCDC39	Atlas Antibodies	HPA035364	1:300	-
Rabbit polyclonal anti-GAS8	Atlas Antibodies	HPA041311	1:500	-
Rabbit polyclonal anti-DNALI1	Atlas Antibodies	HPA028305	1:500	-
Rabbit polyclonal anti-DNAH1	Atlas Antibodies	HPA036805	1:50	1:500
Rabbit polyclonal anti-DNAH6	Atlas Antibodies	HPA036391	1:400	1:500
Rabbit polyclonal anti-DNAH7	Atlas Antibodies	HPA034724	1:200	1:500
Goat anti-mouse Alexa Fluor 488	Thermo Fischer Scientific	A11029	1:1000	1:1000
Goat anti-rabbit Alexa Fluor 546	Thermo Fischer Scientific	A11035	1:1000	1:1000
goat anti-rabbit HRP	GE Healtcare Life Sciences	NA934	-	1:3000
Hoechst33342	Sigma-Aldrich	14533-100MG	1:1000	

Table S2. Table presents genes, which are included in targeted PCD gene panel sequencing.

Genes
ARMC4 (NM_001290020, NM_018076), *CCDC39* (NM_181426), *CCDC40* (NM_017950, NM_001330508, NM_001243342), *CCDC65* (NM_033124), *CCDC103* (NM_213607), *CCDC114* (NM_144577), *CCDC151* (NM_145045), *CCNO* (NM_021147), *CFAP43* (NM_025145), *CFAP44* (NM_001164496), *CFAP45* (NM_012337), *CFAP52* (NM_145054), *CFAP53* (NM_145020), *CFAP69* (NM_001160138, NM_001039706), *CFAP70* (NM_001367801), *CFAP74* (NM_001304360), *CFAP221* (NM_001271049), *CFAP298/C21orf59* (NM_001350335, NM_021254), *CFAP300/C11orf70* (NM_032930), *DNAAF1* (NM_178452, NM_001318756), *DNAAF2* (NM_018139), *DNAAF3* (NM_001256714), *DNAAF4* (NM_130810, NM_001033560), *DNAAF5* (NM_017802), *DNAAF6/PIH1D3* (NM_001169154, NM_173494), *DNAAF11/LRRC6* (NM_012472, NM_001321965), *CFTR* (NM_000492), *DNAH1* (NM_015512), *DNAH2* (NM_020877; NM_001303270), *DNAH5* (NM_001369), *DNAH8* (NM_001206927), *DNAH9* (NM_001372), *DNAH10* (NM_207437), *DNAH11* (NM_001277115), *DNAI1* (NM_001281428), *DNAI2* (NM_001353167), *DNAJB13* (NM_153614), *DNAL1* (NM_031427, NM_001201366), *DRC1/CCDC164* (NM_145038), *DYNC2H1* (NM_001080463), *ENKUR* (NM_145010, NM_001270383), *FOXJ1* (NM_001454), *FSIP2* (NM_173651), *GAS2L2* (NM_139285), *GAS8/GAS11* (NM_001481), *HYDIN* (NM_001270974, NM_001198542), *INVS* (NM_014425), *LRRC56* (NM_198075), *MCIDAS* (NM_001190787), *MNS1* (NM_018365), *NEK10* (NM_152534, NM_001304384), *OFD1* (NM_003611, NM_001330210), *RPGR* (NM_000328, NM_001034853), *RSPH1* (NM_080860), *RSPH3* (NM_031924), *RSPH4A* (NM_001010892), *RSPH9* (NM_001193341), *SPAG1* (NM_003114, NM_172218), *SPEF2* (NM_024867), *STK36* (NM_015690), *TP73* (NM_005427, NM_001126240, NM_001204192), *TTC12* (NM_001318533, NM_001352038, NR_147891), *TTC25* (NM_031421), *TXNDC3/NME8* (NM_016616), *ZMYND10* (NM_015896)

Table S3. Clinical characteristics of individuals with pathogenic bi-allelic variants in *CCDC39* (NM_181426). F: female; M: male; consang: consanguinity; n.a.: not available.

Patient-ID	Sex	Gene	Alteration 1		Alteration 2		Con-sang.	Situs	TEM
F-651 II1	F	*CCDC39*	c.1874G>T[1]	-	c.1874G>T[1]	-	no	SI	n.a.
F-745 II1	F	*CCDC39*	c.2159-2A>G	-	c.1485dup	p.(Ser496Ilefs*15)	yes	SI	n.a.
F-745 II4	F	*CCDC39*	c.2159-2A>G	-	c.1485dup	p.(Ser496Ilefs*15)	yes	SS	n.a.
F-745 II6	M	*CCDC39*	c.2159-2A>G	-	c.1485dup	p.(Ser496Ilefs*15)	yes	SI	tubular dis-organisation
F-901 II0	M	*CCDC39*	c.931-8A>G	-	c.931-8A>G	-	yes	SS	n.a.
F-901 II1	F	*CCDC39*	c.931-8A>G	-	c.931-8A>G	-	yes	SI	n.a.
OI-5 II1	F	*CCDC39*	c.1410G>T	p.(Glu471*)	c.1410G>T	p.(Glu471*)	yes	SI	n.a.
OP-122	M	*CCDC39*	c.1072del	p.(Thr358Glnfs*)	c.485dup	p.(Tyr163Valfs*6)	no	SI	n.a.
OP-336 II1	F	*CCDC39*	c.1036-2A>G	-	c.1036-2A>G	-	no	SS	n.a.
OP-567	M	*CCDC39*	c.643A>T	p.(Lys215*)	c.643A>T	p.(Lys215*)	n.a.	n.a.	tubular dis-organisation
OP-736		*CCDC39*	c.351+1G>C	-	c.2551G>T	p.(Glu851*)	n.a.	SI	n.a.
OP-964 II1	M	*CCDC39*	c.1874G>T[1]	-	c.1252dup	p.(Ala418Glyfs*7)	no	SS	n.a.
OP-1482	F	*CCDC39*	c.610-2A>G	-	c.610-2A>G	-	no	SA	tubular dis-organisation
OP-1867 II1	F	*CCDC39*	c.1874G>T[1]	-	c.216_217del	p.(Cys73Glnfs*6)	no	SS	n.a.
OP-2263 II1	M	*CCDC39*	Deletion Exon 2-6	-	Deletion Exon 2-6	-	yes	SI	tubular dis-organisation
OP-2263 II3 (II2)	F	*CCDC39*	Deletion Exon 2-6	-	Deletion Exon 2-6	-	yes	SS	tubular dis-organisation
OP-2624 II1	F	*CCDC39*	c.1874G>T[1]	-	c.1874G>T[1]	-	no	SI	n.a.
OP-2690 II1	F	*CCDC39*	c.1874G>T[1]	-	c.526_527del	p.(Leu176Alafs*)	n.a.	SI	n.a.

[1] facultative splice site variant (see Figure S2)

Table S4. Overview of the IF stainings in individuals with *CCDC39* variants and allele frequency of the identified *CCDC39* variants. N.a.: not available.

Patient ID	Variant	Allele frequency	IF staining						
			**CCDC39**	**GAS8**	**DNALI1**	**DNAH1**	**DNAH6**	**DNAH7**	**DNAH5**
**F-651 II1**	c.1874G>T	6.50e-5	abnormal	abnormal	abnormal	abnormal	abnormal	abnormal	n.a.
	c.1874G>T	6.50e-5							
**F-745 II1**	c.2159-2A>G	0	n.a.	n.a.	n.a.	n.a.	n.a.	n.a.	n.a.
	c.1485dup; p.(Ser496Ilefs*15)	0							
**F-745 II4**	c.2159-2A>G	0	n.a.	n.a.	n.a.	n.a.	n.a.	n.a.	n.a.
	c.1485dup p.(Ser496Ilefs*15)	0							
**F-745 II6**	c.2159-2A>G	0	n.a.	n.a.	n.a.	n.a.	n.a.	n.a.	n.a.
	c.1485dup p.(Ser496Ilefs*15)	0.							
**F-901 II0**	c.931-8A>G	0	abnormal	abnormal	abnormal	n.a.	abnormal	abnormal	normal
	c.931-8A>G	0							
**F-901 II1**	c.931-8A>G	0	abnormal	abnormal	abnormal	n.a.	n.a.	abnormal	normal
	c.931-8A>G	0							
**OI-5 II1**	c.1410G>T p.(Glu471*)	0	n.a.	n.a.	n.a.	abnormal	n.a.	abnormal	n.a.
	c.1410G>T p.(Glu471*)	0							
**OP-122**	c.1072del p.(Thr358Glnfs*)	3.25e-5	abnormal	abnormal	abnormal	abnormal	abnormal	abnormal	normal
	c.485dup p.(Tyr163Valfs*6)	0							
**OP-336 II1**	c.1035-2A>G	4.06e-5	abnormal	abnormal	abnormal	abnormal	abnormal	abnormal	normal
	c.1035-2A>G	4.06e-5							
**OP-567**	c.643A>T p.(Lys215*)	0	n.a.	n.a.	n.a.	n.a.	n.a.	n.a.	n.a.
	c.643A>T p.(Lys215*)	0							
**OP-736**	c.357+1G>C	1.47e-4	n.a.	n.a.	abnormal	abnormal	n.a.	abnormal	normal
	c.2551G>T p.(Glu851*)	0							
**OP-964 II1**	c.1874G>T	6.50e-5	abnormal	abnormal	abnormal	n.a.	n.a.	n.a.	normal
	c.1252dup p.(Ala418Glyfs*7)	0							
**OP-1482**	c.610-2A>G	1.18e-4	abnormal	n.a.	n.a.	n.a.	n.a.	n.a.	normal
	c.610-2A>G	1.18e-4							
**OP-1867 II1**	c.1874G>T	6.50e-5	abnormal	abnormal	abnormal	n.a.	n.a.	n.a.	normal
	c.216_217del p.(Cys73Glnfs*6)	6.34e-6							
**OP-2263 II1**	Deletion Exon 2-6	0	abnormal	abnormal	n.a.	n.a.	n.a.	n.a.	normal
	Deletion Exon 2-6	0							
**OP-2263 II3 (II2)**	Deletion Exon 2-6	0	abnormal	abnormal	n.a.	n.a.	n.a.	n.a.	normal
	Deletion Exon 2-6	0							
**OP-2624 II1**	c.1874G>T	6.50e-5	abnormal	abnormal	n.a.	n.a.	n.a.	n.a.	normal
	c.1874G>T	6.50e-5							
**OP-2690 II1**	c.1874G>T	6.50e-5	abnormal	abnormal	n.a.	n.a.	n.a.	n.a.	normal
	c.526_527del; p.(Leu176Alafs*10)	1.62e-5							

Table S5. Clinical characteristics of individuals with pathogenic bi-allelic variants in *CCDC40* (NM_017950). F: female; M: male; consang.: consanguinity; n.a.: not available.

Patient-ID	Sex	Gene	Alteration 1		Alteration 2		Con-sang.	Situs	TEM
F-678 II2	F	*CCDC40*	c.248del	p.(Ala83Valfs*84)	c.248del	p.(Ala83Valfs*84)	no	SS	tubular dis-organisation
F-727 II1	F	*CCDC40*	c.248del	p.(Ala83Valfs*84)	c.248del	p.(Ala83Valfs*84)	n.a.	SI	n.a.
OI-101	F	*CCDC40*	Deletion Exon 4-7	-	Deletion Exon 4-7	-	yes	n.a.	tubular dis-organisation
OP-57	F	*CCDC40*	c.1951C>T	p.(Gln651*)	c.1951C>T	p.(Gln651*)	yes	SI	
OP-82 II1	M	*CCDC40*	c.248del	p.(Ala83Valfs*84)	c.1810C>T	p.(Gln604*)	no	SI	n.a.
OP-120	F	*CCDC40*	c.248del	p.(Ala83Valfs*84)	c.248del	p.(Ala83Valfs*84)	n.a.	SS	n.a.
OP-246 II1	M	*CCDC40*	c.248del	p.(Ala83Valfs*84)	c.2457del	p.(Ile819Metfs*15)	n.a.	SI	tubular dis-organisation
OP-277 II1	M	*CCDC40*	c.2824_2825insTGT	p.(Arg942MetinsTrp)	c.3129del	p.(Phe1044Serfs*34)	n.a.	SS	n.a.
OP-307 II2	M	*CCDC40*	c.1345C>T	p.(Arg449*)	c.2825_2826insCTGT	p.(Arg942Thrfs*56)	yes	SI	tubular dis-organisation
OP-472 II1	M	*CCDC40*	c.248del	p.(Ala83Valfs*84)	c.248del	p.(Ala83Valfs*84)	no	SS	n.a.
OP-472 II2	M	*CCDC40*	c.248del	p.(Ala83Valfs*84)	c.248del	p.(Ala83Valfs*84)	no	SS	n.a.
OP-780	M	*CCDC40*	c.248del	p.(Ala83Valfs*84)	c.248del	p.(Ala83Valfs*84)	n.a.	n.a.	tubular dis-organisation
OP-792 II5	M	*CCDC40*	c.248del	p.(Ala83Valfs*84)	c.248del	p.(Ala83Valfs*84)	no	SI	n.a.
OP-852	F	*CCDC40*	c.248del	p.(Ala83Valfs*84)	c.2835+4A>G	-	n.a.	n.a.	tubular dis-organisation
OP-862	M	*CCDC40*	c.248del	p.(Ala83Valfs*84)	c.3097A>T	p.(Lys1033*)	n.a.	n.a.	tubular dis-organisation
OP-1072 II1	F	*CCDC40*	Deletion Exon 1-2	-	Deletion Exon 1-2	-	yes	SS	tubular dis-organisation
OP-1186	F	*CCDC40*	c.2440C>T	p.(Arg814*)	c.2440C>T	p.(Arg814*)	no	SI	tubular dis-organisation
OP-1261 II1	F	*CCDC40*	c.2630del	p.(Glu878Argfs*9)	c.2630del	p.(Glu878Argfs*9)	yes	SS	tubular dis-organisation
OP-1263	M	*CCDC40*	c.248del	p.(Ala83Valfs*84)	c.2182_2183del	p.(Gly728Alafs*26)	n.a.	n.a.	n.a.

OP-1475 II1	M	*CCDC40*	c.1345C>T	p.(Arg449*)	c.3175C>T	p.(Arg1059*)	no	SI	tubular dis-organisation
OP-1753 II1	F	*CCDC40*	c.248del	p.(Ala83Valfs *84)	c.248del	p.(Ala83Valfs *84)	no	SI	tubular dis-organisation
OP-1753 II2	M	*CCDC40*	c.248del	p.(Ala83Valfs *84)	c.248del	p.(Ala83Valfs *84)	no	SS	n.a.
OP-1753 II3	M	*CCDC40*	c.248del	p.(Ala83Valfs *84)	c.248del	p.(Ala83Valfs *84)	no	SS	n.a.
OP-1794	M	*CCDC40*	c.248del	p.(Ala83Valfs *84)	c.2712-1G>T	-	n.a.	n.a.	n.a.
OP-1807 II4	M	*CCDC40*	c.940-1G>C	-	c.940-1G>C	-	yes	SI	n.a.
OP-1854 II1	F	*CCDC40*	c.93+1G>A	-	c.1345C>T	p.(Arg449*)	no	SS	n.a.
OP-1890	M	*CCDC40*	c.248del	p.(Ala83Valfs *84)	c.248del	p.(Ala83Valfs *84)	n.a.	n.a.	n.a.
OP-2072 II2	F	*CCDC40*	c.62C>T	p.(Gly21Val)	c.62C>T	p.(Gly21Val)	yes	SS	tubular dis-organisation
OP-2072 II4	F	*CCDC40*	c.62C>T	p.(Gly21Val)	c.62C>T	p.(Gly21Val)	yes	SS	tubular dis-organisation
OP-2083	M	*CCDC40*	c.248del	p.(Ala83Valfs *84)	c.2182_2183 del	p.(Gly728Alaf s*26)	n.a.	n.a.	n.a.
OP-2242 II1	F	*CCDC40*	c.901C>T	p.(Arg301*)	c.2408del	p.(Lys803Arg fs*13)	no	SS	n.a.
OP-2302 II1	M	*CCDC40*	c.901C>T	p.(Arg301*)	c.2408del	p.(Lys803Arg fs*13)	no	SS	tubular dis-organisation
OP-2407	F	*CCDC40*	c.1345C>T	p.(Arg449*)	c.2712-1G>A	-	no	SI	n.a.

Table S6. Overview of the IF stainings in individuals with *CCDC40* variants and allele frequency of the identified *CCDC40* variants. N.a.: not available

Patient-ID	Variant	Allele frequency	CCDC39	GAS8	DNALI1	DNAH1	DNAH6	DNAH7	DNAH5
F-678 II2	c.248del; p.(Ala83Valfs*84)	5.63e-4	abnormal	abnormal	abnormal	n.a.	n.a.	n.a.	normal
	c.248del; p.(Ala83Valfs*84)	5.63e-4							
F-727 II1	c.248del; p.(Ala83Valfs*84)	5.63e-4	n.a.	abnormal	abnormal	abnormal	abnormal	abnormal	normal
	c.248del; p.(Ala83Valfs*84)	5.63e-4							
OI-101	Deletion exon 4-7	0	abnormal	abnormal	abnormal	abnormal	abnormal	abnormal	normal
	Deletion exon 4-7	0							
OP-57	c.1951C>T; p.(Gln651*)	1.59e-6	n.a.	abnormal	n.a.	abnormal	n.a.	abnormal	normal
	c.1951C>T; p.(Gln651*)	1.59e-6							
OP-82 II1	c.248del; p.(Ala83Valfs*84)	5.63e-4	abnormal	abnormal	abnormal	abnormal	n.a.	abnormal	normal
	c.1810C>T; p.(Gln604*)	0							
OP-120	c.248del; p.(Ala83Valfs*84)	5.63e-4	n.a.	abnormal	abnormal	abnormal	n.a.	n.a.	normal
	c.248del; p.(Ala83Valfs*84)	5.63e-4							
OP-246 II1	c.248del; p.(Ala83Valfs*84)	5.63e-4	abnormal	abnormal	abnormal	n.a.	n.a.	n.a.	normal
	c.2457del; p.(Ile819Metfs*15)	0							
OP-277 II1	c.2824_2825insTGT; p.(Arg942MetinsTrp)	0	abnormal	abnormal	abnormal	abnormal	abnormal	abnormal	normal
	c.3129del; p.(Phe1044Serfs*34)	0							
OP-307 II2	c.1345C>T; p.(Arg449*)	0	abnormal	n.a.	n.a.	n.a.	n.a.	n.a.	normal
	c.2825_2826insCTGT; p.(Arg942Thrfs*56)	0							
OP-472 II1	c.248del; p.(Ala83Valfs*84)	5.63e-4	abnormal	abnormal	abnormal	n.a.	n.a.	n.a.	normal
	c.248del; p.(Ala83Valfs*84)	5.63e-4							
OP-472 II2	c.248del; p.(Ala83Valfs*84)	5.63e-4	abnormal	abnormal	abnormal	n.a.	abnormal	n.a.	normal
	c.248del; p.(Ala83Valfs*84)	5.63e-4							
OP-780	c.248del; p.(Ala83Valfs*84)	5.63e-4	n.a.	abnormal	abnormal	abnormal	abnormal	abnormal	n.a.
	c.248del; p.(Ala83Valfs*84)	5.63e-4							
OP-839	c.248del; p.(Ala83Valfs*84)	5.63e-4	abnormal	abnormal	abnormal	n.a.	n.a.	n.a.	normal
	c.248del; p.(Ala83Valfs*84)	5.63e-4							
OP-852	c.248del; p.(Ala83Valfs*84)	5.63e-4	abnormal	abnormal	abnormal	abnormal	abnormal	abnormal	normal
	c.2835+4A>G	0							
OP-862	c.248del; p.(Ala83Valfs*84)	5.63e-4	abnormal	abnormal	abnormal	abnormal	abnormal	abnormal	normal
	c.3097A>T; p.(Lys1033*)	0							
OP-1072 II1	Deletion Exon 1-2	0	abnormal	abnormal	abnormal	abnormal	abnormal	abnormal	normal
	Deletion Exon 1-2	0							
OP-1186	c.2440C>T; p.(Arg814*)	0	abnormal	abnormal	abnormal	abnormal	abnormal	abnormal	normal
	c.2440C>T; p.(Arg814*)	0							
OP-1261 II1	c.2630del; p.(Glu878Argfs*9)	0	abnormal	abnormal	abnormal	abnormal	abnormal	abnormal	normal
	c.2630del; p.(Glu878Argfs*9)	0							
OP-1263	c.248del; p.(Ala83Valfs*84)	5.63e-4	abnormal	abnormal	abnormal	abnormal	abnormal	abnormal	normal

	c.2182_2183del; p.(Gly728Alafs*26)	0							
**OP-1475 II1**	c.1345C>T; p.(Arg449*)	0	abnormal	abnormal	abnormal	abnormal	abnormal	abnormal	normal
	c.3175C>T; p.(Arg1059*)	0							
**OP-1753 II1**	c.248del; p.(Ala83Valfs*84)	5.63e-4	abnormal	abnormal	abnormal	abnormal	abnormal	abnormal	normal
	c.248del; p.(Ala83Valfs*84)	5.63e-4							
**OP-1753 II2**	c.248del; p.(Ala83Valfs*84)	5.63e-4	abnormal	abnormal	n.a.	n.a.	n.a.	n.a.	normal
	c.248del; p.(Ala83Valfs*84)	5.63e-4							
**OP-1753 II3**	c.248del; p.(Ala83Valfs*84)	5.63e-4	abnormal	abnormal	n.a.	n.a.	n.a.	n.a.	normal
	c.248del; p.(Ala83Valfs*84)	5.63e-4							
**OP-1794**	c.248del; p.(Ala83Valfs*84)	5.63e-4	abnormal	abnormal	n.a.	n.a.	n.a.	n.a.	normal
	c.2712-1G>T	0							
**OP-1854 II1**	c.93+1G>A	0	abnormal	abnormal	n.a.	n.a.	n.a.	n.a.	normal
	c.1345C>T; p.(Arg449*)	0							
**OP-1890**	c.248del; p.(Ala83Valfs*84)	5.63e-4	abnormal	abnormal	n.a.	n.a.	n.a.	n.a.	normal
	c.248del; p.(Ala83Valfs*84)	5.63e-4							
**OP-2072 II2**	c.62C>T; p.(Gly21Val)	0	abnormal	abnormal	n.a.	n.a.	n.a.	n.a.	normal
	c.62C>T; p.(Gly21Val)	0							
**OP-2072 II4**	c.62C>T; p.(Gly21Val)	0	abnormal	abnormal	abnormal	abnormal	n.a.	n.a.	normal
	c.62C>T; p.(Gly21Val)	0							
**OP-2083**	c.248del; p.(Ala83Valfs*84)	5.63e-4	abnormal	abnormal	n.a.	n.a.	n.a.	n.a.	normal
	c.2182_2183del; p.(Gly728Alafs*26)	0							
**OP-2242 II1**	c.901C>T; p.(Arg301*)	0	abnormal	abnormal	n.a.	n.a.	n.a.	n.a.	normal
	c.2408del; p.(Lys803Argfs*13)	0							
**OP-2302 II1**	c.901C>T; p.(Arg301*)	0	abnormal	abnormal	n.a.	n.a.	n.a.	n.a.	normal
	c.2408del; p.(Lys803Argfs*13)	0							
**OP-2407**	c.1345C>T; p.(Arg449*)	0	abnormal	abnormal	n.a.	n.a.	n.a.	n.a.	normal
	c.2712-1G>A	0							
